# A scalable, hyperstable intelligent fibre velocimeter for dynamic digitization of resistance training

**DOI:** 10.1093/nsr/nwaf560

**Published:** 2025-12-11

**Authors:** Jingyu Ouyang, Pan Li, Yuqi Zou, Guangcong Liu, Hongtao Zeng, Rui Han, Duo Li, Weitao Zheng, Jingbo Sun, Guangming Tao

**Affiliations:** Research Center for Intelligent Fiber Devices and Equipment, State Key Laboratory of New Textile Materials and Advanced Processing, School of Physical Education, Wuhan National Laboratory for Optoelectronics, School of Materials Science and Engineering, Department of Geriatrics, Department of Orthopedics, and Key Laboratory of Vascular Aging, Ministry of Education, Tongji Hospital of Tongji Medical College, Huazhong University of Science and Technology, Wuhan 430074, China; State Key Laboratory of Space Medicine, China Astronaut Research and Training Center, Beijing 100094, China; Center for Intelligent Health Interdisciplinary Science, Central China Normal University, Wuhan 430079, China; Center for Intelligent Health Interdisciplinary Science, Central China Normal University, Wuhan 430079, China; Research Center for Intelligent Fiber Devices and Equipment, State Key Laboratory of New Textile Materials and Advanced Processing, School of Physical Education, Wuhan National Laboratory for Optoelectronics, School of Materials Science and Engineering, Department of Geriatrics, Department of Orthopedics, and Key Laboratory of Vascular Aging, Ministry of Education, Tongji Hospital of Tongji Medical College, Huazhong University of Science and Technology, Wuhan 430074, China; Research Center for Intelligent Fiber Devices and Equipment, State Key Laboratory of New Textile Materials and Advanced Processing, School of Physical Education, Wuhan National Laboratory for Optoelectronics, School of Materials Science and Engineering, Department of Geriatrics, Department of Orthopedics, and Key Laboratory of Vascular Aging, Ministry of Education, Tongji Hospital of Tongji Medical College, Huazhong University of Science and Technology, Wuhan 430074, China; Key Laboratory of Sports Engineering of General Administration of Sport of China, Wuhan Sports University, Wuhan 430079, China; Key Laboratory of Sports Engineering of General Administration of Sport of China, Wuhan Sports University, Wuhan 430079, China; Key Laboratory of Sports Engineering of General Administration of Sport of China, Wuhan Sports University, Wuhan 430079, China; Research Center for Intelligent Fiber Devices and Equipment, State Key Laboratory of New Textile Materials and Advanced Processing, School of Physical Education, Wuhan National Laboratory for Optoelectronics, School of Materials Science and Engineering, Department of Geriatrics, Department of Orthopedics, and Key Laboratory of Vascular Aging, Ministry of Education, Tongji Hospital of Tongji Medical College, Huazhong University of Science and Technology, Wuhan 430074, China; Research Center for Intelligent Fiber Devices and Equipment, State Key Laboratory of New Textile Materials and Advanced Processing, School of Physical Education, Wuhan National Laboratory for Optoelectronics, School of Materials Science and Engineering, Department of Geriatrics, Department of Orthopedics, and Key Laboratory of Vascular Aging, Ministry of Education, Tongji Hospital of Tongji Medical College, Huazhong University of Science and Technology, Wuhan 430074, China; State Key Laboratory of Space Medicine, China Astronaut Research and Training Center, Beijing 100094, China; Center for Intelligent Health Interdisciplinary Science, Central China Normal University, Wuhan 430079, China

**Keywords:** fibre sensor, velocity measurement, training digitization

## Abstract

Real-time accuracy and continuous dynamic monitoring capability of devices are crucial for the scientific configuration and dynamic modulation of resistance training, such as strength training, rehabilitation and in-orbit training for astronauts. However, developing monitoring devices capable of providing real-time, accurate and dynamic quantification for high-velocity resistance training remains a notable challenge. Here, we present a scalable and hyperstable intelligent fibre velocimeter designed for the digital, real-time and dynamic monitoring of resistance training. By incorporating the fibre velocimeter as a core component, the intelligent resistance band system demonstrates cyclic stability exceeding 120 000 cycles and torsional insensitivity, facilitating hyperstable velocimetry within the 0–2.5 m/s range with an accuracy exceeding 95%. This system is capable of capturing instantaneous training parameters, including velocity, tension and power during cyclic resistance training, as well as performing dynamic evaluations and providing early warnings for overspeed or fatigue. A comparative experiment with and without feedback guidance from the intelligent resistance band system verified that its precise feedback significantly elevates training intensity, explosive performance and movement compliance while reducing injury risk. Given its compact design, real-time sensing and evaluation of highly accurate multidimensional training parameters, and hyperstability, this system potentially advances training digitization and sports intelligence.

## Teaser

A scalable, hyperstable fibre velocimeter enables real-time monitoring of resistance training with velocimetry accuracy exceeding 95%, aiding rehabilitation, strength, and in-orbit training.

## INTRODUCTION

To proactively address the care and treatment pressures of an ageing population, home-based care has gradually become a mainstream elderly care approach [1]. As a common home-based training method, resistance exercise serves as a fundamental strategy for improving athletic performance and overall health [2]. Across all forms of resistance training, including strength training, rehabilitation and in-orbit training for astronauts, accurate control and real-time dynamic adjustment of exercise load are essential for maximizing training efficacy and minimizing injury risk [3–6]. In addition, approximately 60% of acute injuries during training are directly attributable to fatigue [7]. Thus, accurate quantification of fatigue level is equally crucial for mitigating injury risks. This need for precision becomes even more critical in long-duration spaceflight, where microgravity-induced muscle atrophy and bone demineralization accelerate markedly [8]. Even minor inaccuracies in exercise prescription pose the risk of irreversible muscular damage, making real-time and accurate monitoring of high-velocity resistance training under non-gravitational conditions imperative.

Existing velocity monitoring sensors utilizing linear displacement sensors [9,10] or inertial sensors [11–13] enable dynamic monitoring of velocity and power. However, multiple interrelated challenges constrain their effectiveness in high-velocity resistance training under non-gravitational conditions. Among them, linear displacement sensors are restricted to 1 m/s, thereby precluding high-velocity resistance-exercise monitoring, and their output is liable to torsional interference. Inertial sensors exhibit integration-dependent drift and scaling errors that accrue over time, while gravitational coupling further degrades absolute accuracy. To ensure real-time and accurate acquisition of training parameters, velocity monitoring sensors in resistance training face challenges that include providing continuous hyperstable monitoring across a broad range of human motion velocities [14] (i.e. 0–2.5 m/s), resisting torsional effects and remaining independent of gravitational influences.

Adapting to the 1D stretchability of resistance bands, the development of functional fibres [15–17] with scalability, and embedded functional components [17–20] presents a promising approach. Thermal drawing technology [15,21] facilitates structural stability and scalability in fibre integration moulding. Additionally, compared with fibre sensors based on solid conductive composites [22], liquid metal (LM) [23–25], characterized by high conductivity [26] and shape adaptability [27], mitigates sensor instability caused by torsional effects and enhance stretchability and durability, which is critical for monitoring complex, high-degree-of-freedom trajectory motions. The sensing functionality based on fluid deformation caused by shear forces generated during the stretching process is independent of gravitational influences, which further broadens sensor applications to non-terrestrial environments, such as underwater or space.

**Figure 1.**
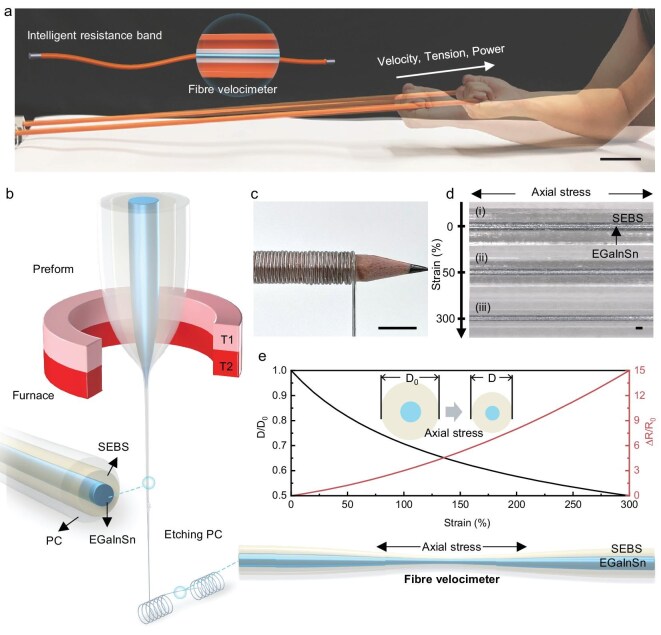
Fabrication and characteristic performance of the fibre velocimeter. (a) Photograph of the movement of the intelligent resistance band during training; scale bar 10 cm. The inset shows the structure of intelligent resistance band. (b) Schematic of the thermal drawing technique for fabricating the fibre velocimeter. (c) Flexibility of the fibre velocimeter; scale bar 1 cm. (d) Transmission optical micrographs of fibre velocimeters captured at three different strains: (i) initial fibre velocimeter; (ii) 50% strain; and (iii) 300% strain; scale bar 100 μm. (e) Rate of change in diameter and resistance of a fibre velocimeter with increasing strain according to the theoretical model.

To address the multifaceted challenges of velocity monitoring sensors, we present a new paradigm based on fibre velocimeters whose LM core is fully encapsulated within an elastomeric sheath and acts as the flowing conductor. This design is expected to provide continuous monitoring hyperstability and gravity independence, even under high-velocity and torsional conditions. Upon axial elongation of the fibre, the sheath transmits shear to the LM, inducing cross-sectional reduction and a corresponding increase in electrical resistance. According to Ohm’s law, the tensile velocity is linearly related to the time-derivative of the square root of the flowing conductor’s resistance (see Methods). Consequently, the stretching velocity of the fibre can be derived from resistance and time data. By incorporating the fibre velocimeter as its core component, the intelligent resistance band system enables dynamic, real-time and hyperstable digitization of resistance training (Video S1), achieving velocimetry accuracy exceeding 95% (Fig. 1a). Integration of a constitutive model that relates the resistance band’s elongation to tension, together with advanced data visualization techniques, extends the system’s capability to capture instantaneous physical parameters, including velocity, tension and power during cyclic resistance training. This facilitates the dynamic assessment of muscle fatigue and overspeed with timely alerts issued. To evaluate its versatility and hyperstability, the device was tested in multiple scenarios, including rehabilitation, strength training and astronaut muscle training in microgravity.

## RESULTS AND DISCUSSION

### Fibre velocimeter design and fabrication

Fibre velocimeters, which actively convert velocity into electrical signals, are manufactured using a versatile thermal drawing [28] method that allows for scalable production. The process involves two primary steps: (i) preparing a coaxial preform (Fig. S1) and (ii) drawing the preform into fibres (Fig. 1b). To achieve optimal elastic response and electrical conductivity, thermoplastic elastomer styrene ethylene butylene styrene (SEBS) was selected as the outer stretchable layer, while LM (Ga_68.5_/In_21.5_/Sn_10_, EGaInSn) was utilized as the inner conductive layer, forming an intrinsically stretchable sensor [22]. EGaInSn offers the best compromise among room-temperature liquidity, high conductivity, controllable oxidation and cost-effectiveness. SEBS simultaneously fulfils the key requirements of super-elasticity, low modulus, fatigue durability, drawability and biocompatibility, making it the optimal sheath material for the LM fibre velocimeter. To maintain stability during the drawing process, a sacrificial layer of polycarbonate (PC) was applied to the outer surface of the preform and subsequently removed following fibre drawing. The fabricated fibre velocimeter exhibits a core-sheath structure, comprising a smooth LM core enclosed within a circular SEBS sheath (Fig. S1e). Owing to the shape adaptability of LM alloys and the high stretchability of SEBS, the fibre velocimeter demonstrates exceptional flexibility, allowing it to be uniformly coiled around a pencil (Fig. 1c). By modifying feed speed and drawing parameters, the fibre diameter can be precisely regulated, facilitating continuous production (Fig. S1f).

The theoretical model suggests that the LM, maintaining a constant volume within the fibre, flows in response to external shear forces induced by fibre stretching (Fig. 1d). This flow results in the elongation of the liquid column and a reduction in its cross-sectional area, leading to an increase in resistance (Fig. 1e). To accommodate the 1D stretchability of resistance bands, an intelligent resistance band was developed by integrating the fibre velocimeter into a commercially available hollow resistance band. Utilizing the closed microchannel fluid tensile velocimetry model, real-time resistance variations occurring during training can be transformed into parameters such as velocity, facilitating its application in resistance exercise scenarios.

### Characterization of fibre velocimeter

**Figure 2.**
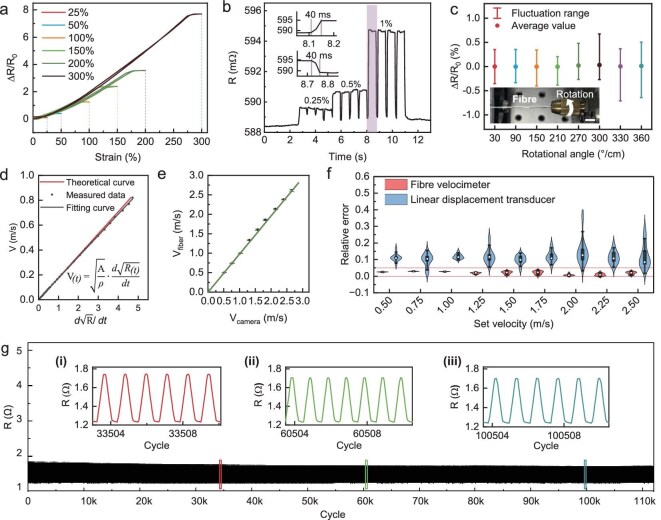
The characteristic performance of fibre velocimeter. (a) Hysteresis contraction of the fibre velocimeter within strain levels of 25%, 50%, 100%, 150%, 200% and 300%. (b) Electrical response of the fibre velocimeter at 0.25%, 0.5% and 1% strain, with response and recovery times of 40 ms. (c) Resistance change rate remains below 0.8% across the entire range from 30°/cm to 360°/cm; scale bar 1 cm. (d) Linear relationship between the change rate of root resistance and velocity, with a linear correlation coefficient of 99.96%. (e) Correlation between fibre velocimeter and high-speed camera for synchronous velocity measurement. (f) The relative error distribution of the fibre velocimeter within the velocity range of 0–2.5 m/s is consistently within 5%, which is far superior to that of the linear displacement sensor. (g) Mechanical durability of the fibre velocimeter over 120 000 cycles at 50% strain, with details.

The electrical response characteristics of the fibre velocimeter were examined under various stretching conditions to assess its velocity-measuring function and applicability in intelligent resistance bands. Due to the constant volume of LM and minimal contact resistance, the relationship between the relative resistance change of the fibre velocimeter and strain follows a non-linear relationship [Supplementary Text, Equation (5)], aligning with the theoretical model (Fig. S2a). The fibre velocimeter also exhibits variable volt–ampere characteristics typical of resistive devices under different strain conditions (Fig. S2b). To evaluate its mechanical and electrical properties, hysteresis contraction was tested within strain levels of 25%, 50%, 100%, 150%, 200% and 300%. The findings demonstrate effective tensile recovery performance up to 300% strain (Fig. 2a) and a fracture elongation of 500% (Fig. S2c). Moreover, the resistance signal response of the fibre velocimeter remains synchronized with actual strain variations, showing no significant drift under sustained strain (Fig. S2d). Additionally, the device exhibits high repeatability and consistency in electrical signals under repeated cycles of stretching and releasing across different strain levels (Fig. S2e). Furthermore, the fibre velocimeter maintains frequency independence when subjected to stretching frequencies ranging from 0.25 to 2 Hz (Fig. S2f).

The response time and strain resolution of fibre velocimeters are key performance indicators. The fibre velocimeter demonstrates a strain resolution of 0.25% and a response time of 40 ms (Fig. 2b). To evaluate the effect of torsion, the fibre velocimeter was twisted from 30°/cm to 360°/cm. The resistance change rate remains within 0.8% under 360° torsion per centimetre (Fig. 2c), confirming torsional insensitivity, which reduces velocity measurement errors during resistance training. In contrast, for applications requiring torsional sensitivity, a spiral weaving structure can enhance the torsional response of the fibre (Fig. S3). Overall, the fibre velocimeter achieves a wide strain range, high resolution, rapid response, and torsional insensitivity, making it well suited for velocity measurement in resistance training applications.

### Working mechanism of fibre velocimeter

Based on the closed microchannel fluid tensile velocimetry model, the LM in the fibre core remains at a constant volume while adapting its shape during stretching. For 1D cylindrical LM in fibre velocimeters, instantaneous velocity can be determined from instantaneous resistance variations using the induction formula (see Methods). To verify the relationship between resistance and velocity, the fibre velocimeter was stretched at varying velocities while real-time resistance measurements were recorded, revealing a linear relationship between the change rate of root resistance and velocity (Fig. 2d). The linear correlation coefficient of 99.96% confirms the feasibility of deriving velocity from resistance variations.

To further validate the accuracy of the fibre velocimeter, it was mounted alongside a linear displacement sensor on a sliding device to ensure consistent testing velocities. The velocity measured by the fibre velocimeter closely aligned with that recorded by a high-speed camera (Fig. 2e). Using the high-speed camera data as a reference (Fig. 2f), the fibre velocimeter exhibited a relative error of less than 4% across the human motion velocity range (0–2.5 m/s) [14], demonstrating superior performance compared to the displacement sensor. Additionally, the fibre velocimeter maintained high repeatability and consistency under repeated stretching at varying velocities (Fig. S4), indicating stable responsiveness. Mechanical durability is essential for long-term applications. The fibre velocimeter sustained over 120 000 cycles at 50% strain (Fig. 2g), over 42 000 stretching cycles at 100% strain (Fig. S5a), over 19 000 stretching cycles at 200% strain (Fig. S5b) and over 6000 stretching cycles at 300% strain (Fig. S5c). These results exceed the performance of other stretching sensors reported in previous studies [29–36] (Fig. S6). Given that human joint stretching typically does not exceed 300% strain based on human body proportions [37], the fibre velocimeter meets the durability requirements necessary for resistance training applications.

### Intelligent resistance band system based on fibre velocimeter

**Figure 3.**
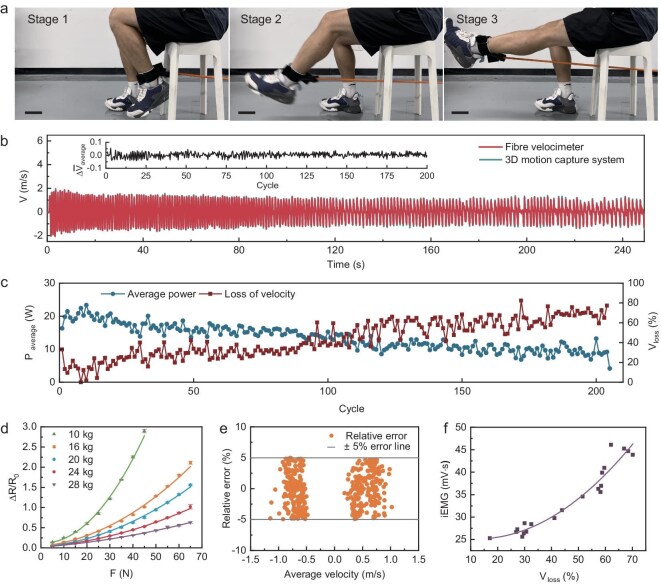
Intelligent resistance band system based on fibre velocimeter. (a) Photograph of leg resistance training using the intelligent resistance band; scale bar 20 cm. (b) Synchronization of leg resistance training velocity measurement using the fibre velocimeter and 3D motion capture system. The inset shows cycle average velocity differences between the fibre velocimeter and 3D motion capture system. (c) Average power and velocity loss rate measured by the fibre velocimeter during leg resistance training. (d) Relationship between tension and resistance change rate for intelligent resistance bands with resistance levels of 10, 16, 20, 24 and 28 kg. (e) Relative error of average velocity measured by intelligent resistance bands compared to 3D motion capture system. (f) Correlation between velocity loss rate and integrated electromyography.

To facilitate the monitoring of resistance training parameters and states, an intelligent resistance band interaction system was developed. The system employs a standard Bluetooth communication method. Initially, the intelligent resistance band is connected to a micro-resistance acquisition chip equipped with Bluetooth communication capabilities to continuously capture the resistance characteristics of the band. The chip transmits data to a computer terminal through Bluetooth, where resistance and time data are processed to derive parameters such as the number of cycles, velocity, average power and velocity loss rate [Supplementary Text, Equations (8)–(14)]. The system’s functionality was demonstrated using leg flexion-extension exercises in a seated position (Fig. 3a). Based on the sensing mechanism of the fibre velocimeter, the square root of the resistance value was derived over time and multiplied by a coefficient to determine real-time velocity. These velocity data were synchronized with measurements obtained from a 3D motion capture system (Fig. 3b), showing an average velocity difference of less than 0.05 m/s (inset of Fig. 3b). The average velocity recorded by the intelligent resistance band exhibited a relative error of less than 5% per cycle compared to the 3D motion capture system (Fig. 3e), confirming the system’s reliability. Similar accuracy was observed in forearm-bending exercises (Fig. S7a–d).

The tension of resistance bands, as a form of progressive resistance, exhibits a non-linear relationship with elongation [Supplementary Text, Equation (13)]. The regression equation [38] for the tension of resistance bands of varying colours under different stretching rates has been analysed. Regression equations for different resistance bands were derived (Fig. 3d). For leg exercises using a 10-kg resistance band, tension was calculated based on the rate of resistance change (Fig. S7e). After the fibre velocimeter is integrated into a band of a specific weight, a single calibration is performed. The circuit module that reads, processes and transmits the resistance signal is detachable. When switching to a band of a different weight, the interface can be unplugged and reconnected to the new band. Average power and velocity loss rate were obtained from stretching velocity and tension data (Fig. 3c). As the number of training cycles increased, average power declined while the velocity loss rate increased. Velocity loss rate [39] [Supplementary Text, Equation (11)] is highly correlated with muscle fatigue indicators [40,41] such as repetition rate, perceived fatigue and blood lactate levels, enabling quantification of muscle fatigue, thereby reducing injury risk and enhancing exercise efficiency. Additionally, velocity loss rate was correlated with surface integrated electromyographic (iEMG) signals (Fig. S7f), yielding a Spearman correlation coefficient of 0.82475 and a *P* value of 1.17538 × 10^−51^, confirming a strong relationship (Fig. 3f).

A visualization interface for resistance training parameters was developed with two modes (Video S2): (i) load and fatigue monitoring, which displays velocity, tension, power and cycle statistics; and (ii) fatigue and overspeed warning, which alerts users to potential muscle injury risks based on monitored velocity and fatigue levels. Upon centrifugal overspeed, the overspeed ratio value immediately turns red. Fatigue trend and load are displayed in real time as bar charts: orange for moderate fatigue and red for high fatigue. All parameters refresh every 0.5 s, enabling users to receive visual alerts directly on the computer terminal.

To demonstrate the broad applicability of the intelligent resistance band, its performance was evaluated across various training scenarios. The flexibility, stretchability and torsional insensitivity of the fibre velocimeter embedded within the band enable real-time and accurate monitoring of multi-plane and multi-joint movements across different training postures. As a 1D, multi-joint exercise, squats require the fibre velocimeter to undergo a deformation rate exceeding 100% during the squatting motion (Fig. S8a). To ensure uniform weight distribution between both ends during squat training, a fibre velocimeter with negligible weight was employed for monitoring. Real-time velocity monitoring of squat training was successfully conducted using the fibre velocimeter (Fig. S8b). For multi-planar movements, the system effectively tracked velocity during anti-rotation exercises (Fig. S8c and d), demonstrating superior performance compared to single-plane high-speed cameras.

**Figure 4.**
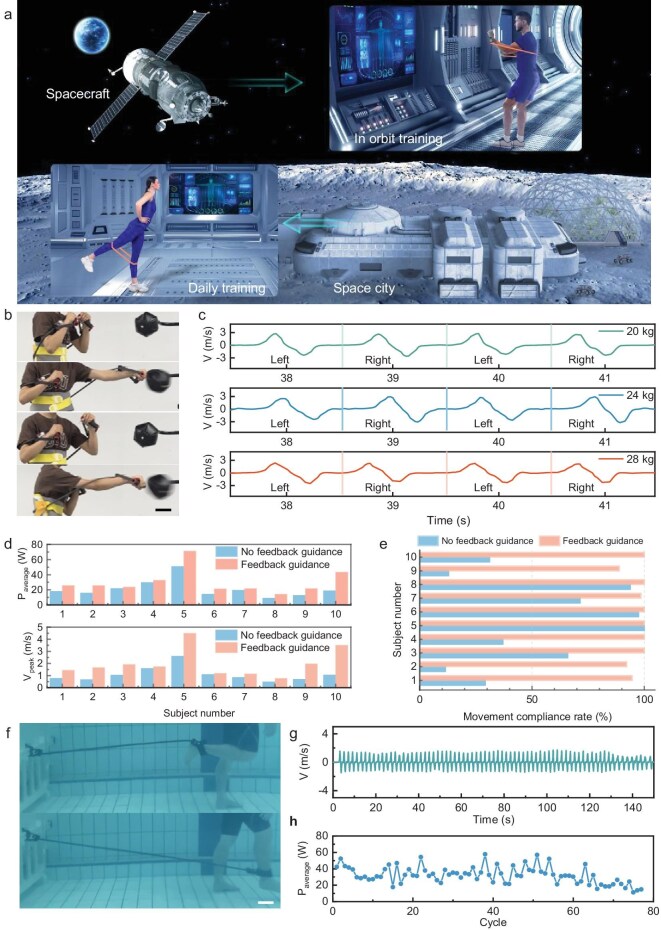
Scalability in function and application of intelligent resistance band system. (a) Schematic illustration of intelligent resistance band system assisting training in space. (b) Upper-limb training based on intelligent resistance band; scale bar 10 cm. (c) Velocity of the left and right arms with intelligent resistance bands at 20, 24 and 28 kg resistance levels. (d) Average power and peak velocity of 10 subjects were measured under two conditions: with and without feedback guidance. (e) Movement compliance rate of 10 subjects was measured under two conditions: with and without feedback guidance. (f) Usability testing of intelligent resistance bands in a microgravity environment through underwater training; scale bar 10 cm. (g) Velocity curve of underwater training with intelligent resistance band. (h) Average power of underwater training with intelligent resistance band.

In the microgravity environment of space, the degradation of astronauts’ anti-gravity muscles poses a significant threat to health and overall functionality [42–44]. As a lightweight and portable training solution unaffected by weightlessness, resistance training utilizing the intelligent resistance band system has become an essential method for maintaining astronauts’ physical health and work capacity in orbit [45–47]. The intelligent resistance band system, incorporating the fibre velocimeter, enables real-time status evaluation and is expected to enhance resistance training strategies in space (Fig. 4a). The intelligent resistance bands with resistance levels of 20, 24 and 28 kg were utilized to assess exercise dynamics for upper-limb training (Fig. 4b). The results indicated that heavier bands led to a more rapid decline in velocity (Fig. S8e) and lower average power (Fig. S8f), while also reducing the number of repetitions completed before reaching subjective fatigue, defined as an 80% velocity loss rate (Fig. S8g). Furthermore, in cases where differences exist in muscle explosiveness and endurance between an individual’s left and right arms, the intelligent resistance band system continuously monitored key parameters such as velocity (Fig. 4c), average power (Fig. S8f) and velocity loss rate of both arms (Fig. S8g). This capability enables the evaluation of asymmetries in muscle strength and endurance, providing an objective basis for symmetrical training on both sides [48].

An investigation was conducted to determine whether the real-time digitization of an intelligent resistance band system provides positive feedback on training efficacy, with data on training parameters collected from 10 subjects using the system. Specifically, the average power, peak velocity and eccentric overspeed occurrences of subjects were measured under two conditions: with and without feedback guidance (Fig. 4d and e). These metrics were employed to evaluate training intensity, explosive performance and movement compliance rate. The findings demonstrated that feedback guidance from the intelligent resistance band system significantly enhanced subjects’ athletic performance, as evidenced by higher average power and peak velocity (Fig. 4d). Additionally, the data on movement compliance rate showed that feedback guidance from the intelligent resistance band system effectively corrected improper movements, thereby minimizing the risk of muscle injuries (Fig. 4e).

A subaquatic rehabilitation training simulation was conducted to investigate the usability of the intelligent resistance-band system within an aquatic therapeutic environment (Fig. 4f and Video S3). Velocity, average power and fatigue metrics were stably monitored with stability (Fig. 4g and h, and Fig. S8h). Underwater training also demonstrates the potential application of the intelligent resistance band in microgravity environments. These findings highlight the system’s adaptability to diverse environments and its potential applications in digital resistance training.

## CONCLUSIONS

This study developed a scalable and hyperstable intelligent fibre velocimeter for digital, real-time and dynamic monitoring of training parameters, establishing a new paradigm for velocity measurement and a novel approach to training digitization. The proposed fibre velocimeter achieves a monitoring error of less than 4% within the velocity range of 0–2.5 m/s, while exhibiting high stretchability, cyclic stability and torsional insensitivity, offering significant advantages in velocity monitoring technology. Additionally, the fibre velocimeter is compatible with resistance bands featuring 1D stretchability, enabling the development of an intelligent resistance band system with velocimetry accuracy over 95%. These intelligent resistance bands are well-suited for practical application across diverse resistance training scenarios. Furthermore, their lightweight and portable design, unaffected by microgravity, makes them particularly advantageous for maintaining astronauts’ health and work performance in space. In the future, the fibre velocimeter is expected to be widely utilized in training monitoring to establish an intelligent exercise prescription library. By integrating historical training data with real-time physiological feedback, this system can dynamically optimize training regimens, significantly advancing the digitization of sports and enhancing the intelligence of training equipment. Additionally, the integration of such intelligent fibre velocimeters into robotic systems could enhance the precision and adaptability of robotic-assisted training and rehabilitation, paving the way for more personalized and effective robotic interventions in the future.

## METHODS

### Fabrication of fibre velocimeter

The fibre velocimeter was fabricated via a thermal-drawing strategy. Figure S1a illustrates the fabrication process of the fibre preform. The primary polymer materials of the preform are commercially available: SEBS (SEBS, G1650, Kraton Co., Ltd.) particles and PC (ET3113, Dongguan Xindayuan Co., Ltd.) particles. Semi-cylindrical SEBS assembly with a groove was produced via a hot-pressing method in a semi-cylindrical mould, which was equipped with a stainless-steel rod, at a temperature of 185°C and an external pressure of 0.3–3 MPa. Two such SEBS assemblies were then combined, with a Teflon rod inserted to prevent deformation during subsequent heat processing. These assemblies were placed in a muffle furnace at 175°C for heat consolidation, resulting in the formation of the hollow SEBS preform (Fig. S1b). PC films, produced via hot-pressing, were wound around the exterior of the hollow preform and heat-consolidated in a vacuum tube furnace at 175°C. By removing the Teflon rod, the hollow PC–SEBS preform was obtained (Fig. S1c). Subsequently, the LM EGaInSn (Ga 68.5%, In 21.5%, Sn 10%; Sino Santech Materials Technology Co., Ltd.) was injected into the hollow hole of the preform and the ends were heat-sealed to create an LM–SEBS–PC multimaterial preform. The preform was drawn into 500-μm-diameter fibre using a custom-built fibre tower at a speed of ∼4 m/min under an applied temperature of 260°C–300°C (Fig. S1d). After thermal drawing, the outer PC sacrificial layer with ∼50 μm thickness was etched using dimethylacetamide (DMAC) with the assistance of ultrasonic cleaner (Fig. S1e).

### Fibre velocimeter model

Starting from the 1D cylindrical conductor structure model of the fibre velocimeter and the volume invariance of the EGaInSn inside the fibre, a reliable formula model has been established between the resistance change rate $\sqrt R $ and the velocity of fibre velocimeters (Supplementary Text). The instantaneous velocity of the fibre velocimeter is calculated as:


(1) \begin{eqnarray*}
&&{V}_{\left( t \right)} = \frac{{d{L}_{\left( t \right)}}}{{dt}} = \sqrt {\frac{A}{\rho }} \cdot \frac{{d\sqrt {{R}_{\left( t \right)}} }}{{dt}} = a \cdot \frac{{d\sqrt {{R}_{\left( t \right)}} }}{{dt}},{\mathrm{\ }}\\
&&\quad a = \sqrt {\frac{A}{\rho }},
\end{eqnarray*}

where *V*_(_*_t_*_)_ represents the instantaneous velocity at which the fibre is stretched, *t* is time, *L* is the length at which the fibre velocimeter is stretched at a certain moment, *R* is the resistance of the fibre velocimeter under a specific tensile stress, *A* is the volume of the conductive liquid metal cylinder inside the fibre velocimeter and *ρ* is the resistivity of the LM in the fibre velocimeter.

### Measurement and calculation of the velocity

A 3D motion capture system (Vicon Motion Systems, Oxford, UK, 200 HZ) was used to measure velocity as reference value during training. For comparison, a linear displacement sensor (Briter Corporation) and our fibre velocimeter were used to measure velocity synchronously. The fatigue status of muscle was also measured by a commercial EMG device (Trigno™ Wireless, Delsys).

## Supplementary Material

nwaf560_Supplemental_Files

## Data Availability

The data in plots generated in this study are provided in the Supplementary data. Custom code and the dataset used in the current study are available in the GitHub database (https://github.com/NSR2025/Intelligent-Resistance-Band).
