# Supplementary material for: A scalable, hyperstable intelligent fibre velocimeter for dynamic digitization of resistance training
Source: Natl Sci Rev. 2025 Dec 11;13(8):nwaf560. doi: 10.1093/nsr/nwaf560 (PMC13142149; doi:10.1093/nsr/nwaf560)
Supplement: nwaf560_Supplemental_Files [file nwaf560_supplemental_files.zip › Supplementary data.pdf]

# Supplementary Materials for

## A Scalable, Hyperstable Intelligent Fibre Velocimeter for Dynamic Digitisation of Resistance Training

Jingyu Ouyang[1,2]*, Pan Li[3]*, Yuqi Zou[3]*, Guangcong Liu[1]*, Hongtao Zeng[1]*, Rui Han[4], Duo Li[4], Weitao Zheng[4], Jingbo Sun[1], Guangming Tao[1,2,3] †

1) Research Center for Intelligent Fiber Devices and Equipment, State Key Laboratory of New Textile Materials and Advanced Processing, School of Physical Education, Wuhan National Laboratory for Optoelectronics, School of Materials Science and Engineering, Department of Geriatrics, Department of Orthopedics, and Key Laboratory of Vascular Aging, Ministry of Education, Tongji Hospital of Tongji Medical College, Huazhong University of Science and Technology, Wuhan 430074, China.

2) State Key Laboratory of Space Medicine, China Astronaut Research and Training Center, Beijing 100094, China.

3) Center for Intelligent Health Interdisciplinary Science, Central China Normal University, Wuhan 430079, China.

4) Key Laboratory of Sports Engineering of General Administration of Sport of China, Wuhan Sports University, Wuhan, 430079, China.

*These authors contributed equally to this work.

†Corresponding author. Email: tao@hust.edu.cn

**The file includes:**

Supplementary Text

Figs. S1 to S6

**Other Supplementary Materials for this manuscript include the following:**

Movies S1 to S3

**Supplementary Text**

Characterization testing. Fibre velocimeters were immersed in liquid nitrogen for several minutes, then immediately clipped with tweezers and its cross sections were observed by optical microscope (CX40M, Shunyu Optical Technology 481 Co., Ltd.). The tensile strength of fibre velocimeters was measured using a mechanical testing machine (Dongguan Zhiqu Precision Instrument Co., Ltd., ZQ-990B). For connection, Cu wires with a diameter of 100 um were inserted into the fibre followed by sealing with epoxy. The tested fibres were stretched with an electric sliding table (RXPN40BJ, Chengdu Liandong Ruixin Technology Corporation) with a control cabinet. The resistance was simultaneously monitored with a digital multimeter (DMM6500, Keithley Instruments Inc.) at a sampling rate of 50 Hz. A resistance-analyzing circuit with an integrated HC-05 Bluetooth module (Zhihe 01RC, Linkzill) was used. The resulting acquisition device measures 68 mm × 68 mm × 20 mm, weighs 60 g, and covers the 0–200 MΩ resistance range. The device conforms to Bluetooth Specification V2.0 With EDR, provides an experimentally measured data-sampling rate of 87.5 Hz, and exhibits a typical transmission latency of ≈ 8 ms under normal wireless-signal conditions.

Intelligent resistance band configuration. An inner diameter hollow commercial resistance band (Sun Frame Corporation, natural-latex formulation) was used as fibre velocimeters packaging substrate. Fibre velocimeters passed through the hollow resistance belt with the assistance of the thin iron wire. Bare copper wires (100 μm diameter) were first used to connect the liquid-metal interface to the detection circuit. To improve interfacial electrical stability, they were later replaced by an insulated copper-core conductor consisting of three 100 μm strands twisted together. The ends of the hollow resistance band were sealed by the hollow Teflon stick with an inner diameter of 2 mm and an outer diameter of 6 cm. To further fix the fibre, the gap between fibre and the Teflon stick was strengthened by UV-curable adhesive (Resin glue, Su Feng Corporation).

Fibre velocimeter model. Starting from the one-dimensional cylindrical conductor structure model of the fibre velocimeter and the volume invariance of the EGaInSn inside the fibre, a reliable formula model has been established between resistance change rate $\sqrt{R}$ and velocity of fibre velocimeters. In the strain sensing of fibre velocimeters, the relative resistance change of the fibre velocimeter is

$$\frac{\Delta R}{R_0} = \frac{R-R_0}{R_0} \quad (1)$$

Among them, $R$ is the resistance of the fibre velocimeter under a specific tensile stress, and $R_0$ is the initial resistance of the fibre velocimeter without stretching, $\Delta R$ is the resistance relative change of the fibre velocimeter.

Tensile stress of fibre velocimeter at different times can be expressed as formula,

$$\varepsilon = \frac{L-L_0}{L_0} \quad (2)$$

where $L$ is the length at which the fibre velocimeter is stretched at a certain moment, and $L_0$ is the initial length of the fibre velocimeter.

The volume A of the conductive liquid metal cylinder inside the fibre velocimeter under different stretching states is constant,

$$A = L \cdot S \quad (3)$$

where $\rho$ is the resistivity of the liquid metal in fibre velocimeters, and $S$ is the cross-sectional area of the liquid metal in fibre.

For one-dimensional cylindrical liquid metal in fibre velocimeters, the resistance of the fibre velocimeter is

$$R = \rho\frac{L}{S} = \rho\frac{L^2}{A} \quad (4)$$

According to formulas (1), (2), (3), and (4), the formula model (5) for the variation of the electrical signal of the fibre velocimeter with stress can be obtained

$$\frac{\Delta R}{R_0} = (\varepsilon^2 + 2\varepsilon) \quad (5)$$

Derived from formula (4), during the stretching process of the fibre velocimeter, the length L of the fibre velocimeter is

$$L_{(t)} = \sqrt{\frac{A}{\rho}} \cdot \sqrt{R_{(t)}} \quad (6)$$

The velocity at which the fibre velocimeter is stretched is the differential of the stretching length over time,

$$V_{(t)} = \frac{dL_{(t)}}{dt} \quad (7)$$

where $t$ is time, $V_{(t)}$ represents the instantaneous velocity at which the fibre is stretched.

According to formulas (6) and (7), the instantaneous velocity of the fibre velocimeter is

$$V_{(t)} = \frac{dL_{(t)}}{dt} = \sqrt{\frac{A}{\rho}} \cdot \frac{d\sqrt{R_{(t)}}}{dt} = a \cdot \frac{d\sqrt{R_{(t)}}}{dt}\ , a = \sqrt{\frac{A}{\rho}} \qquad (8)$$

In order to improve the convenience of using the resistance band, we threaded the fibre from the resistance band terminal to the other end and then back, with both ends of the fibre being led out from the terminal. The fibres have a symmetrical structure inside the resistance band. Therefore, the instantaneous velocity of the fibre velocimeter mentioned above is

$$V_{(t)} = \frac{1}{2}\frac{dL_{(t)}}{dt} = \frac{1}{2}\sqrt{\frac{A}{\rho}} \cdot \frac{d\sqrt{R_{(t)}}}{dt} = b \cdot \frac{d\sqrt{R_{(t)}}}{dt}\ , b = \frac{1}{2}\sqrt{\frac{A}{\rho}} \qquad (9)$$

Fatigue state prediction model. The velocity loss rate variable is defined as the percentage of the difference between each action velocity and the maximum action velocity within the group in a group of exercise training. The average velocity $\bar{V}$ of the motion was used as the velocity calculation variable, which can be expressed as

$$\bar{V} = \frac{\int_{t_1}^{t_2} V dt}{t_2 - t_1} \qquad (10)$$

where $t_1$ and $t_2$ respectively represent the start and end times of the intelligent resistance band being stretched in one stretching cycle, and $V$ is the measured velocity of the intelligent resistance during being stretched. The average velocity of multiple stretching movements in a set of training can be regarded as a set of velocity $\overline{V}_i$ and the maximum of average velocity $\overline{V}_{max} = Max\{\bar{V}_1, \bar{V}_2, \bar{V}_3, ..., \bar{V}_n\}$, where it represents the average velocity of all movement cycles in a set of stretching exercises. The intelligent resistance band was used to predict the fatigue state of the human body using the formula

$$V_{loss} = \frac{\overline{V}_i}{\overline{V}_{max}} \qquad (11)$$

where $V_{loss}$, $\overline{V}_i$, and $\overline{V}_{max}$ are the rate of velocity loss, the mean velocity, and the maximum mean velocity during exercise respectively.

Tension resistance relationship model. Based on the regression equations for the tension of different colored tensioners at different strain, and the relationship between tension $F$ and strain is approximately as follows:

[1] $$F = m + k\varepsilon \qquad (12)$$

Among them, $m$ and $k$ are coefficients, which are related to the weight of the resistance band. Based on equation (5), there is a nonlinear coupling relationship between the internal resistance change rate and tension of the resistance band:

$$\frac{\Delta R}{R_0} = cF^2 + dF + e \quad (13)$$

Among them, $c$ , $d$ and $e$ are coefficients, which are related to the weight of the resistance band. Based on the acquired tension and real-time speed, the average cyclic power $\overline{\mathrm{P}}$ can be obtained.

$$\overline{\mathrm{P}} = \frac{\sum_{t_1}^{t_2} \mathrm{F} \cdot \mathrm{v}}{t_2 - t_1} \quad (14)$$

Where $t_1$ is the start time of the drawing action within the resistance exercise cycle, and $t_2$ is the stop time of the forward stretch.

<u>The relative error of average velocity.</u> The relative error in the article is calculated over the entire resistance-training process. It compares the average velocity of the concentric phase in each cycle measured by the fibre velocimeter $\overline{V}_{fiber}$ with that measured by the high-speed camera $\overline{V}_{cam}$ .

$$\overline{V}_{fiber} = \frac{\int_{t_1}^{t_2} V_{fiber} dt}{t_2 - t_1} \quad (15)$$

$$\overline{V}_{cam} = \frac{\int_{t_1}^{t_2} V_{cam} dt}{t_2 - t_1} \quad (16)$$

The relative error is

$$\text{error} = \frac{\overline{V}_{fiber} - \overline{V}_{cam}}{\overline{V}_{cam}} \quad (17)$$

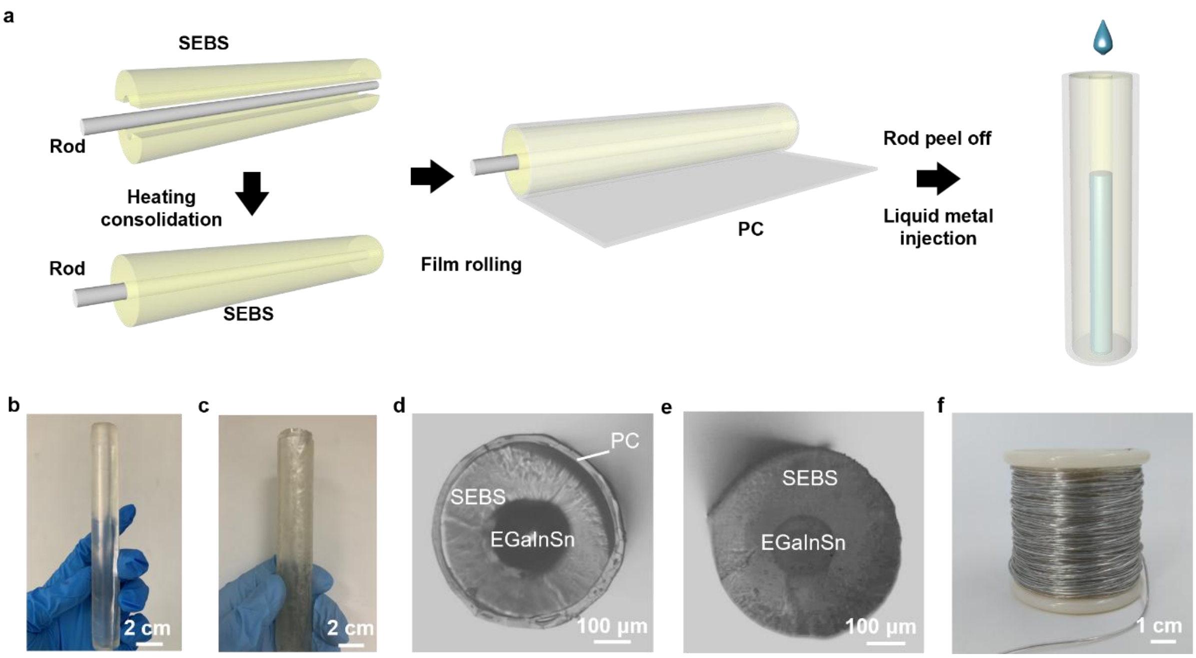


**Supplementary Figure 1.** Fabrication of the fibre velocimeter. (a) Schematic of the fibre velocimeter preparation. (b) Hollow SEBS preform. (c) Hollow PC-SEBS preform. (d) Optical micrograph of LM-SEBS-PC fibre section. (e) Optical cross-section view of fibre velocimeter. (f) Photographs showing scalable fibre velocimeter fabricated by thermal drawing.

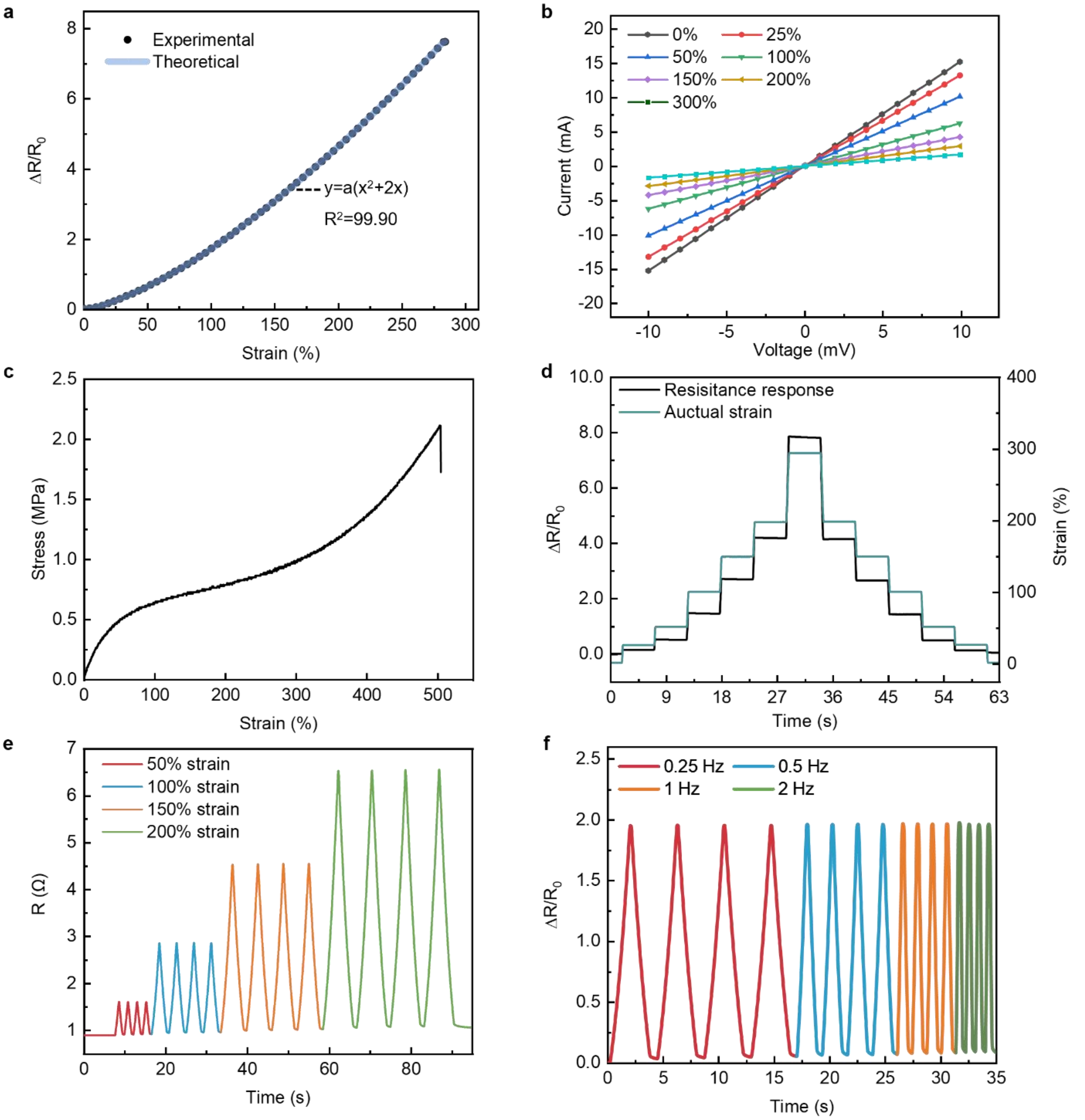


**Supplementary Figure 2.** Characteristic of fibre velocimeters. (a) Relationship between relative resistance change of the fibre velocimeter and strain. (b) Variation of volt-ampere characteristics curve of resistive devices under different strains. (c) The tensile fracture curve of the fiber velocimeter. (d)Synchronous response curve of resistance and strain of the fiber velocimeter at different stages of deformation maintenance. (e)Resistance response of fiber velocimeters under repeated stretching and releasing. (f)Frequency resistance response curve of the fiber velocimeter under 100% strain.

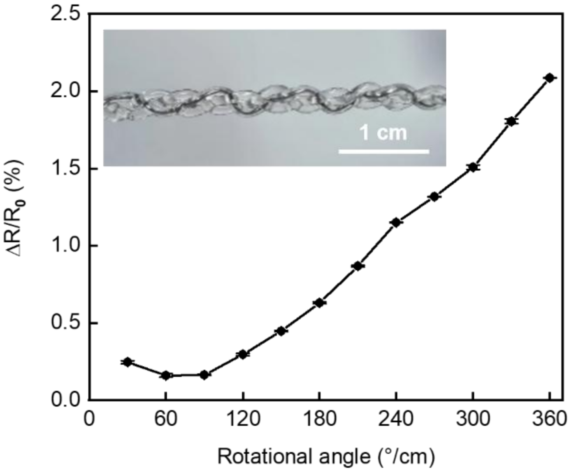


**Supplementary Figure 3.** Torsional sensitivity of a knot sensor composed of a fiber velocimeter and two elastic fibers.

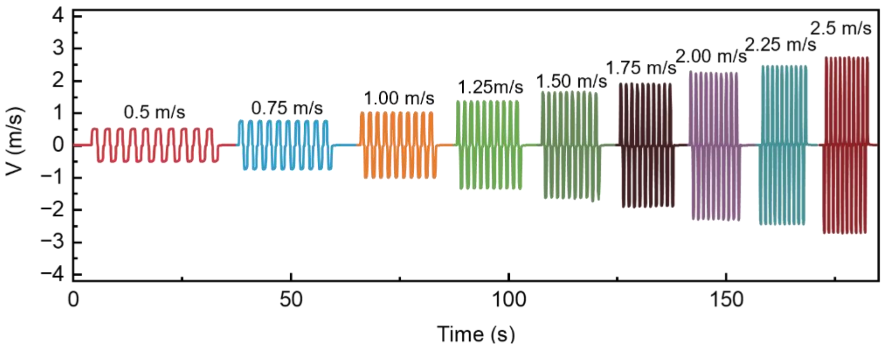


**Supplementary Figure 4.** Repetitive consistency of the fibre velocimeter across the motion velocity range of 0–2.5 m/s.

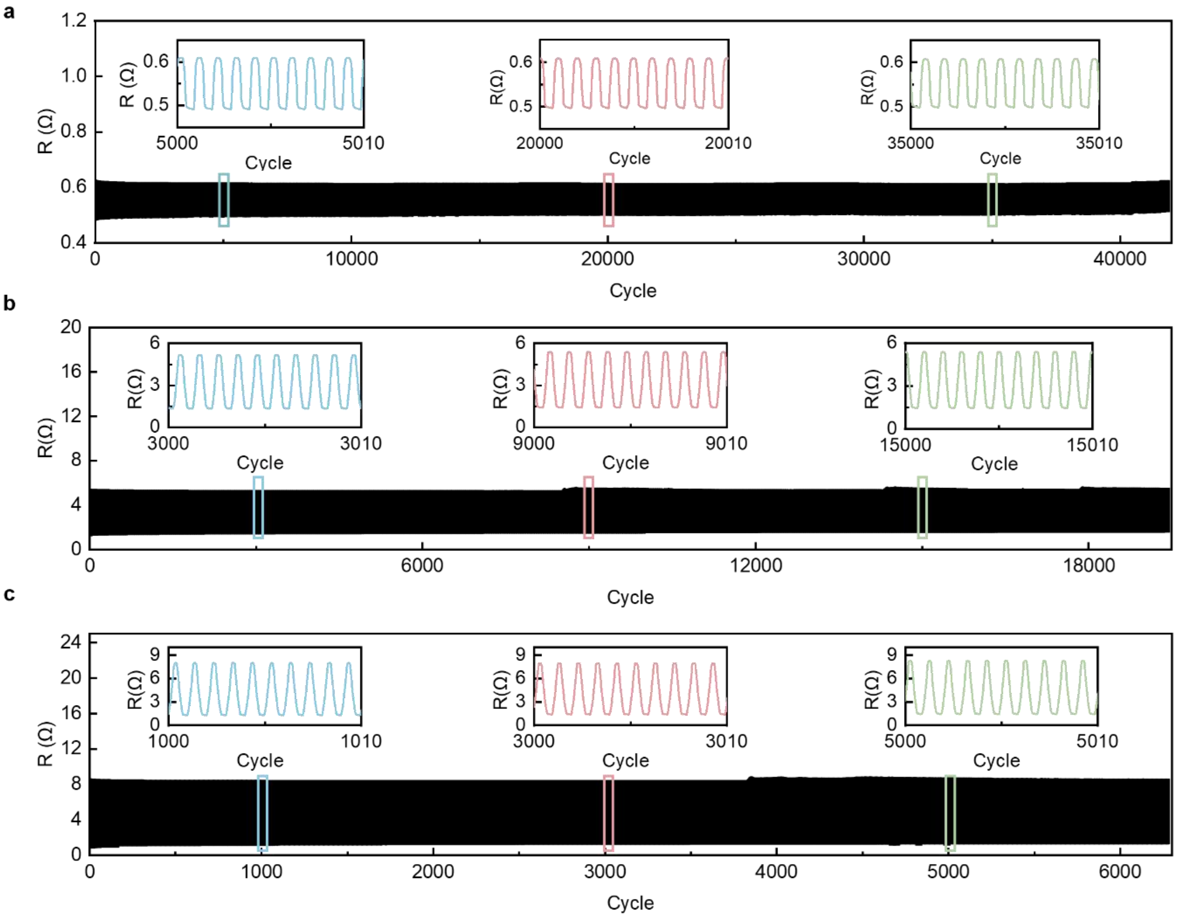


**Supplementary Figure 5.** Durability of fibre velocimeters. (a) The stable performance of the fibre velocimeter over 42,000 stretching cycles at 100% strain. (b) The stable performance of the fibre velocimeter over 19,000 stretching cycles at 200% strain. (c) The stable performance of the fibre velocimeter over 6000 stretching cycles at 300% strain.

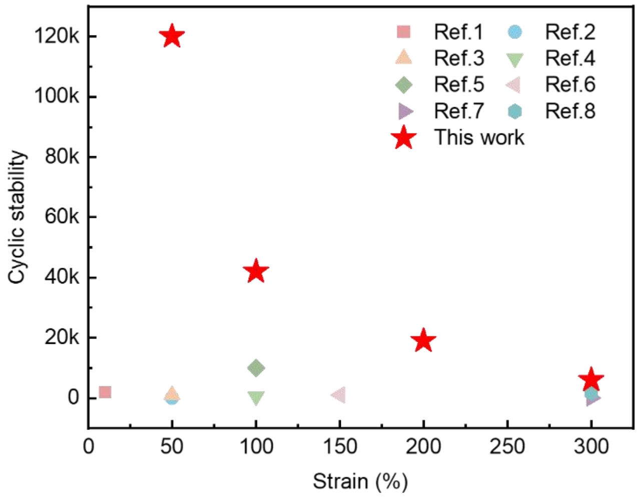


**Supplementary Figure 6.** Comparison of the device cycle stability with other works.

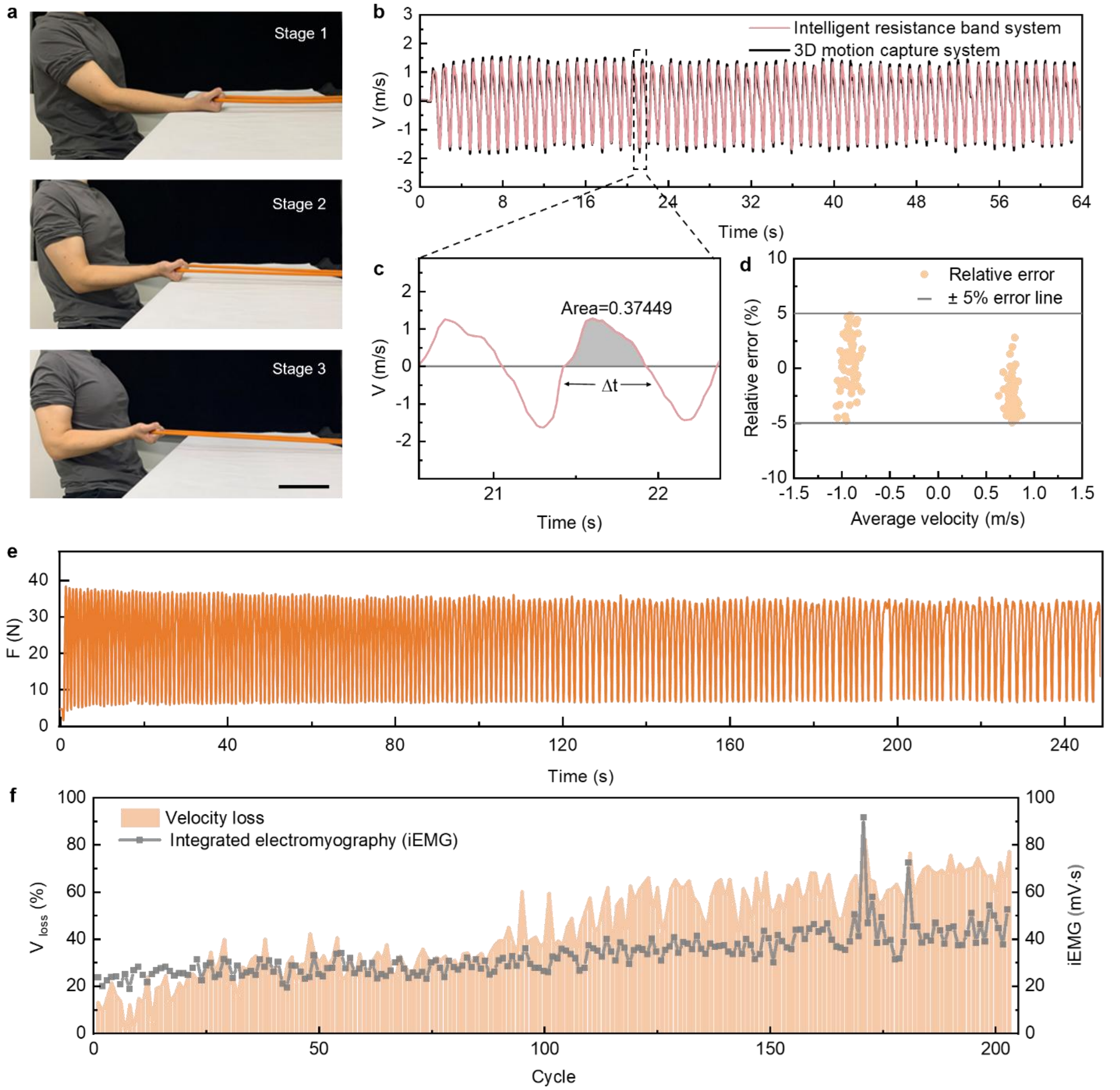


**Supplementary Figure 7.** Accuracy of velocity and fatigue monitoring based on intelligent resistance bands. (a) Forearm bending training based on intelligent resistance band, scale bar 10 cm. (b) Velocity measured by the fibre velocimeter and 3D motion capture system during forearm bending training. (c) Detail view of velocity curve measured by the fibre velocimeter. (d) Relative error between the average velocity of the intelligent resistance band and 3D motion capture system measured per cycle for forearm bending training. (e) Tension measured by the fibre velocimeter during leg resistance training. (f) Synchronous measurement of velocity loss rate and muscle surface integrated electromyography (iEMG) during leg flexion and extension training.

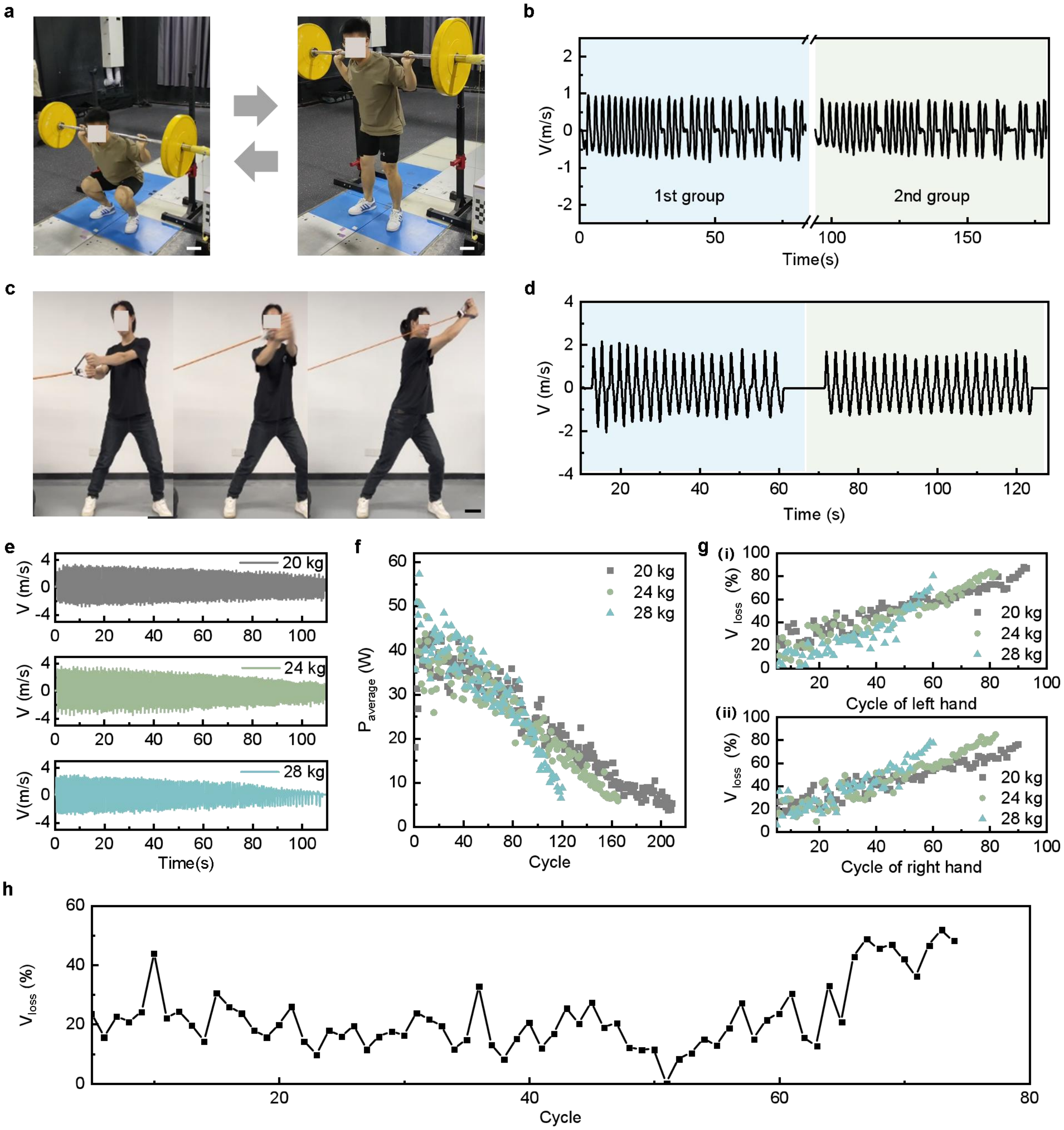


**Supplementary Figure 8.** Expandable application scenarios of fibre velocimeter. (a) Fibre velocimeter for squat training, scale bar 10 cm. (b) Velocity of squat training measured by fibre velocimeter. (c) The intelligent resistance band for anti-rotation training, scale bar 10 cm. (d) Velocity for anti-rotation training measured by fibre velocimeter. (e) Real time velocity of upper limb training with intelligent resistance bands of different weights such as 20 kg, 24 kg, and 28 kg. (f) Average power of the same person under different weight intelligent resistance band of 20 kg, 24 kg, and 28 kg during the same frequency periodic upper limb training. (g) Relationship between the number of cycles and the rate of velocity loss of (i) left hand and (ii) right hand in

upper limb training with different weights. (h) Relationship between the number of cycles and the rate of velocity loss of underwater training.

**Supplementary Video 1.**
A concise overview of this article

**Supplementary Video 2.**
Intelligent resistance band system for digitisation of resistance training

**Supplementary Video 3.**
Usability of intelligent resistance bands in underwater microgravity environment
